# Brief Group Intervention Using Emotional Freedom Techniques for Depression in College Students: A Randomized Controlled Trial

**DOI:** 10.1155/2012/257172

**Published:** 2012-07-17

**Authors:** Dawson Church, Midanelle A. De Asis, Audrey J. Brooks

**Affiliations:** ^1^Foundation for Epigenetic Medicine, 3340 Fulton Road, No. 442, Fulton, CA 95439, USA; ^2^College of Science, University of Santo Tomas, Manila, Philippines; ^3^Department of Psychology, University of Arizona, Tucson, AZ, USA

## Abstract

Two hundred thirty-eight first-year college students were assessed using the Beck Depression Inventory (BDI). Thirty students meeting the BDI criteria for moderate to severe depression were randomly assigned to either a treatment or control group. The treatment group received four 90-minute group sessions of EFT (Emotional Freedom Techniques), a novel treatment that combines exposure, cognitive reprocessing, and somatic stimulation. The control group received no treatment. Posttests were conducted 3 weeks later on those that completed all requirements (*N* = 18). The EFT group (*n* = 9) had significantly more depression at baseline than the control group (*n* = 9) (EFT BDI mean = 23.44, SD = 2.1 versus control BDI mean = 20.33, SD = 2.1). After controlling for baseline BDI score, the EFT group had significantly less depression than the control group at posttest, with a mean score in the “nondepressed” range (*P* = .001; EFT BDI mean = 6.08, SE = 1.8 versus control BDI mean = 18.04, SE = 1.8). Cohen's *d* was 2.28, indicating a very strong effect size. These results are consistent with those noted in other studies of EFT that included an assessment for depression and indicate the clinical usefulness of EFT as a brief, cost-effective, and efficacious treatment.

## 1. Introduction

Depression is a common condition in teenagers. A review by the National Institutes of Health (NIH) found that some 20% of adolescents suffer from bouts of anxiety and depression before they reach adulthood [1]. Adolescent mood disorders are associated with the maturation process, with the stress associated with physiological changes, with the influence of changing hormone levels, with ambivalence toward increased independence, and with adjustments in their relationships with parents and siblings. Depression may also be a reaction to a disturbing event, such as, the death of a friend or relative, a problem in a peer or family relationship, or failure at school. According to the National Comorbidity Survey, prevalence rates for major depression are consistently found to be higher in younger individuals than older ones. Depressive episodes generally last for about 8 months; over 8% of adolescents suffer from depression that lasts a year or more, compared to 5.3% of the general population [1].

Biologically, depression is associated with reduced availability in the brain of the neurotransmitter serotonin, with a decrease in the volume of the hippocampus and the prefrontal complex, and with increased activity of the right prefrontal cortex [2, 3]. Adolescents who have low self-esteem are highly self-critical and who feel little sense of control over negative events are particularly at risk of depression when they experience stressful events. Depression puts adolescents at risk for abusing drugs and alcohol as they may use these substances to self-medicate [4]. The biological, social, and mental health sequelae of adolescent depression makes effective treatment of this condition a high priority in mental health settings.

EFT (Emotional Freedom Techniques) is one of a group of therapies collectively referred to as “energy psychology” or EP. EFT has been shown to be efficacious for depression in a number of studies, including three randomized controlled trials (RCTs). Brattberg [5] assessed depression in a sample of 36 fibromyalgia patients undergoing an 8-week online EFT course. She found a significant reduction in depressive symptoms (*P* < .02). A second RCT, this time examining a population of 59 war veterans who received six sessions of EFT, also found a significant drop in depression, with scores going from clinical to subclinical levels (*P* < .0001) [6]. Participants maintained their gains on followup. An RCT of teenagers treated with EFT for traumatic memories found that the experimental group experienced significant reduction of emotional triggering and a return to normal values on the assessments, while the untreated control group did not improve over time [7]. A hospital in Britain's National Health Service (NHS) conducted an RCT comparing Eye Movement Desensitization and Reprocessing (EMDR) with EFT in 46 patients with clinical PTSD. It found that both EFT and EMDR were efficacious in four sessions [8]. The NHS study also collected data on depression and found both therapies to significantly improve depressive symptoms in the same four-session time frame (*P* = .006).

Several outcome studies employing within-subjects designs found similar results. One examined the effects of a day-long EFT workshop delivered in group format to 216 healthcare workers [9]. It found a clinically and statistically significant drop in depressive symptoms (*P* < .0001). A pilot study of veterans with comorbid PTSD and depression found a significant reduction in depression after six EFT sessions (*P* < .001) [10]. Another pilot study with veterans who received a weeklong intensive treatment found posttest scores for depression significantly lower than at pretest (*P* < .005) [11]. A study by Rowe [12] found that a two-day group EFT seminar significantly reduced depression in the 102 participants assessed (*P* < .0001). Rowe's [12] study was replicated with several other groups, with similar significant results [9, 13]. In all these studies, participants maintained their improvements during follow-up periods of between 3 months and 2 years. RCTs of EFT for test anxiety in college students have also shown significant symptom improvements [14, 15]. The current study is unique in that, unlike the demographic profiles in other studies that have assessed depression, it examines the efficacy of EFT in an adolescent population.

A review of evidence-based PTSD treatments by the Institute of Medicine found that successful psychotherapies have two ingredients in common: exposure and cognitive reprocessing [16]. EFT contains elements of both exposure and cognitive therapy but to these established methods, it adds the novel element of somatic stimulation. A typical EFT session has the client recall a traumatic incident. The memory is then rated on a Likert-type scale from 0 to 10, with 0 being no emotional intensity and 10 being the maximum emotional intensity. This scale is referred to as SUD, Subjective Units of Distress [17]. The emotional memory is then paired with a statement of self-acceptance, for example, “Even though I saw my mother throw the lamp at my father during their argument, and I was very scared…” (exposure), “…I deeply and completely accept myself” (cognitive shift). The client may, for example, self-assess the emotional intensity of a troubling event at a level of 8 out of 10. While keeping the incident in mind, the client adds a somatic stimulus, in the form of touching or tapping a prescribed series of acupressure points. After the process, the client then rerates the intensity of the trauma on the 0 to 10 scale. If the number is still high, EFT is applied again, till the SUD reaches a low number. Each application of EFT takes only a few minutes, but clients typically report a rapid diminution of emotional triggering associated with the traumatic memory. EFT originated in the early 1990s when Stanford-trained engineer Gary Craig simplified an earlier energy psychology (EP) method called Thought Field Therapy or TFT [18]. It is practiced with a high degree of uniformity because *The EFT Manual *[19] has been available as a free online download since the inception of the method in 1995.

Besides quickly reducing affect, therapists have noted a lack of abreactions in clients using EFT [20–22]. For these reasons, therapists report that EFT and other EP methods are a preferred treatment when dealing with traumatized clients [23]. In a therapy session, a client may use EFT on a series of emotionally triggering memories; even traumas of long duration are found to reduce in intensity, often to the surprise of the client and therapist [24]. The parsimony of treatment required to treat PTSD, depression, and other psychological conditions has been noted in other studies; even complex clinical PTSD may resolve in four to six EFT sessions [8, 10].

Feinstein [25] reviewed the success of brief EP treatments in resolving emotional trauma during natural and human-caused disasters. A single session of cognitive restructuring, paired with exposure, can significantly reduce PTSD symptoms [26]. Protocols that introduce a somatic component may be more effective in reducing effect than those that do not [27–30]. During exposure to emotionally troubling memories, Feinstein [24] notes that acupressure reinforces cognitive restructuring, as well as having a calming effect on the client.

Acupressure point stimulation has been the subject of numerous brain imaging studies. Hui et al. [31] found that acupuncture sends signals directly to the amygdala and other structures in the brain's limbic system that process fear. This work has been confirmed by others [32–34]. Fang et al. [35] states that acupuncture produces “extensive deactivation of the limbic-paralimbic-neocortical system.” Acupressure, in which pressure is applied to acupoints, instead of the more familiar insertion of acupuncture needles, has been found to be as effective as needling [36].

The physiological mechanisms of action of EFT have been elucidated in a number of studies. Church et al. [37] conducted a randomized controlled trial of 83 patients that measured cortisol levels before and after treatment with either EFT, psychotherapy, or relaxation. They found that EFT significantly reduces cortisol levels when compared with the other two treatments. Moreover, reductions in cortisol were significantly correlated with depression, anxiety, and other psychological conditions. Diepold and Goldstein [38] tested the brain wave frequencies associated with fear before and after EP treatment. They found that when a traumatic memory is recalled, these frequencies are activated. After EP treatment, they normalize and even on later followup, the recall of the traumatic memory by the client does not reactivate them.

Other studies using qEEG have found that EFT normalizes brain function in traumatized patients [39–41]. Feinstein [27] reviewed published EP research, summarizing its psychological and physiological aspects, and found that it “quickly and permanently reduces maladaptive fear responses to traumatic memories and related cues.” Lane [42] reviewed the literature on the application of EP acupoint stimulation as a counterconditioning method in psychotherapy. He describes physiological mechanisms consistent with a lowering of the stress response and a calming of the threat-assessment structures in the midbrain. These include a reduction in the body's secretion of stress hormones, such as, cortisol, an increase in endogenous opioids, and a dampening of fear in the amygdala. This body of evidence provides a rationale for this study, which examined the use of EFT for the treatment of depression in adolescent college students.

## 2. Method

### 2.1. Participants

For the current study, all 238 first-year BS Psychology students enrolled in the College of Science at the University of Santo Tomas, Manila were assessed using the Beck Depression Inventory. Thirty met the inclusion criterion of having scores in the moderate to severe clinical range (described below). They were randomly assigned to either an EFT or a no treatment group; since the entire cohort was assessed, those with clinical scores had a theoretically equal opportunity of being assigned to either group. The causes of depression listed by participants were personal appearance, romantic relationships, family problems, academic problems, socioeconomic status, and loss of a loved one. To make the results as generalizable as possible for this population, there were no exclusion criteria.

The study was reviewed for human subject protection by the university and registered on http://www.clinicaltrials.gov/ (NCT01117532); all subjects signed informed consent forms. All EFT instruction was provided by a student trained in EFT who served as group facilitator. All assessments and the intervention took place at a location near the College of Science campus. Data were analyzed by a blind offsite biostatistician (the third author). EFT was administered according to the protocols in *The EFT Manual* [19]. The first author, who has an EFT Cert-1 certification from EFT founder Gary Craig, as well as a CEHP credential from ACEP, the Association for Comprehensive Energy Psychology, reviewed implementation fidelity by means of written session notes. Randomization and group assignment were conducted by the second author.

### 2.2. Measures

The Beck Depression Inventory (BDI) is composed of 21 questions that assess the intensity, severity, and depth of depression in patients with psychiatric diagnoses [43]. Items are rated on a 4-point scale, with higher scores indicating more severe depression. Total scores less than 10 are considered no or minimal depression, while scores ranging between 10 and 18 are considered mild depression. Moderate depression scores range between 19 and 29, while severe depression is indicated by scores greater than 29. The BDI has convergent validity with observer-rated measures diagnosing depression [44, 45]. It has been found to be an effective screening instrument for depression in adolescents [46].

### 2.3. Procedures

**Figure 1:**
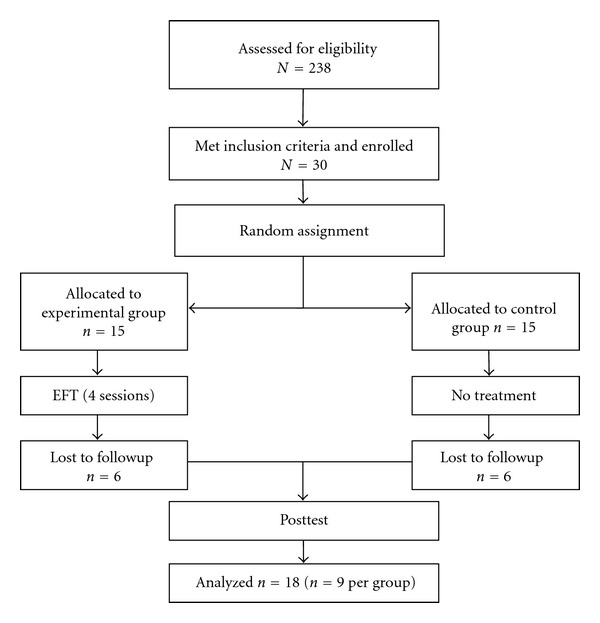
CONSORT flow chart.

Four EFT group therapy sessions were administered within 3 consecutive weeks. Each session lasted 90 minutes. Subjects were dropped from the study if they missed more than one session (*n* = 2 for EFT group) or failed to compete the final assessment (*n* = 4 for EFT group, *n* = 6 for the no treatment group). Posttests for both groups occurred at the end of the 3-week period. Dropouts resulted in a final count of 18 students, and all analysis was performed on this sample. The demographic characteristics of the sample were as follows: 3 were male, and 15 were female; the average age was 16.7 years old, with a range from 16 to 18. No adverse events were reported. The reasons cited by dropouts were lack of time, conflicts with academic requirements, the pressures of exams and class assignments, and forgetting a required group class or assessment completion meeting. See Figure 1 for the CONSORT flow chart.

## 3. Results

First, a *t*-test was conducted to compare baseline BDI scores between the two groups to determine initial equivalence between the groups. A statistically significant difference between groups (*t*(16) = 2.749, *P* = .014) was found indicating greater depression in the EFT experimental group (see Table 1). Due to the lack of equivalence between the two groups, an analysis of covariance (ANCOVA) controlling for baseline scores was conducted on the posttest BDI scores. A statistically significant group effect was found (*F*(1,15) = 18.79, *P* = .001). The EFT group had significantly lower BDI depression scores (see Table 2) at posttest.

The clinical significance of these findings is that, while participants in both groups scored in the moderate-severe range for depression at pretest, the BDI values at posttest for the EFT group improved to place mean participant scores in the nondepressed range. Cohen's *d* was calculated and found to be 2.28. This large effect size is consistent with other EFT studies in which a value of  .8 or above has been found for clinical outcome measures.

## 4. Discussion

This study provides initial evidence that a brief group EFT program might be an efficacious treatment for depression in this high-risk demographic group. The reductions in depressive symptoms reported here are consistent with the results of the studies cited above that examine depression levels in adults. Treating depression in adolescents has inherent clinical value. Depressive episodes in adolescents result in a high risk of subsequent depression [47]. Depression in adolescents is associated with an increased risk of affective disorders in adulthood, as well as an elevated risk of psychiatric treatment and hospitalization in adults [48]. Adolescent depressive symptoms predict dysphoria as adults, as well as behaviors, such as, smoking, prescription drug use, illicit drug use, phobias, suicide, and an inability to establish close intimate relationships [49, 50].

As well as the problems that individuals with depression may experience, the cost of depression to society is high. A review of the depression cost analyses published from 1970 to 1998 found that depression costs the USA economy $65 billion a year in 1998 prices [51]. Each depressed adult worker is estimated to cost his or her employer $1,800 per year [52]. The effect of untreated mental illness is not confined to the individual; the entire community in which they live can be affected [53]. For these reasons, quick and efficacious methods of treating adolescent depression can pay dividends to both individuals and society for decades subsequent to treatment and are thus highly cost effective.

There are several limitations to this study. One is the lack of an active comparison group. An intervention, such as, cognitive behavior therapy, which has demonstrated efficacy for depression when delivered to groups, would test EFT against another evidence-based treatment, as well as controlling for expectancy effects, and the nonspecific effects of any treatment. A second limitation is the lack of a follow-up data point to determine if participant gains were maintained over time. In an EP research review, Feinstein [54] notes that in all studies of EFT that included a follow-up assessment, participants maintained their gains, with the duration of rehabilitation assessed between 3 months and 2 years. An extension of the present study that includes one or more follow-up assessments would determine if this effect is noted for depression in adolescents. A third limitation is the lack of a formal *DSM-IV* diagnosis of depression using observer-rated measures. Though the BDI has demonstrated convergent validity with observer-rated measures [44, 46], an extension of this study should include a clinical diagnosis of depression by a licensed mental health professional. A final limitation to this study is the nonequivalence of depressive symptoms in the two groups. This might indicate that random assignment might be unable to provide significance in an *N* this small, though an ANCOVA was used to adjust for the difference. The higher levels of depressive symptoms in the EFT group on pretest might have made them more motivated to improve.

## 5. Research Recommendations

We speculate that other factors might play a role in the efficacy of EFT delivered to adolescents. One such factor is the level of training of the EFT provider. In the current study, the EFT intervention was delivered by a student with only an introductory level of training in EFT. It is possible that providers with greater training, such as, licensed mental health professionals specializing in group therapy and the treatment of major depressive disorder, might achieve better results. Professionals might utilize methods, drawn from their clinical experience, that are beyond the scope of practice of a novice EFT provider, to produce deeper symptom reductions. Professionals might also perceive subtle therapeutic cues from participants that are missed by nonprofessionals. It is also possible that these advantages may be offset to some degree by the type of peer-to-peer delivery used in this study, which reduces the power differential between the group facilitator and the participants. The high attrition rate among participants is typical of this demographic and is enhanced by diagnosis with a mood disorder [55]. It might be reduced in future studies by allowing participants to miss more than one group session or by collecting data from all available subjects regardless of compliance to the protocol.

Second, we hypothesize that more sessions of EFT might produce a greater effect. The research plan for the current study originally called for *N* = 70 and 12 sessions but faculty skepticism concerning the efficacy of tapping acupressure points led to the reduction of both by more than half and to the elimination of multiple assessments and a follow-up data point. Cognitive dissonance at the speed of resolution of psychological problems when EFT is employed is common and has been noted as a major barrier to the acceptance of EP therapies in the mental health profession [24].

Third, delivery of EFT in groups should be compared with individual therapy sessions, in order to determine which is more efficacious. Other studies have noted efficacy for depression symptoms in EFT group therapy [9, 12]. Nonspecific factors present in individual counseling, such as, sympathetic attention and therapist allegiance, might produce greater symptom reductions; alternately, the power of shared experience might reinforce the gains obtained by group treatment. Self-application between group sessions might further decrease symptoms, as EFT is used to reduce the emotional triggering of everyday situations as they arise. Additionally, the cost effectiveness of group application argues for empirical evaluation of its efficacy as compared with individual treatment sessions.

## 6. Conclusions

In the current study, 238 first-year psychology students were assessed for depression using the Beck Depression Inventory (BDI). Thirty were found to have scores indicative of moderate to severe depression, were enrolled in the study, and randomly assigned to either an EFT group or a no treatment control group. After four 90-minute group EFT sessions, the treatment group demonstrated a clinically and statistically significant improvement in their depression scores, with a mean in the “nondepressed” range, while the control group did not show a comparable reduction in depression scores. These results are consistent with other published reports indicating that brief courses of group EFT are efficacious in treating depression. Further research is required to determine whether self-reports correlate with observer-rated measures, how EFT compares with an active control, whether individual sessions produce greater effects than group sessions, whether EFT is as effective as mental health treatment when delivered as peer counseling, whether the results hold over time, and whether more sessions of EFT produce a greater effect.

## Figures and Tables

**Table 1:** Pretest BDI means and standard deviations: *t*-test Results.

Group	*N*	Mean ± SD
EFT	9	23.44 ± 2.7
NT	9	20.33 ± 2.1

**Table 2:** Posttest BDI means and standard error controlling for Pretest, ANCOVA Results.

Group	*N*	Mean ± SE
EFT	9	6.08 ± 1.8
NT	9	18.04 ± 1.8
